# Self-collected dried blood spots for phosphatidylethanol assessment in virtual trials: Feasibility and concordance with self-reported alcohol use among men at high HIV risk

**DOI:** 10.21203/rs.3.rs-10541536/v1

**Published:** 2026-08-03

**Authors:** Tyler Wray, Erik Ocean, Abhishek Aggarwal, Christopher Kahler, Philip Chan, Chanda Phelan, Peter Monti

**Affiliations:** Brown University; Brown University; Brown University; Brown University; Brown University; Brown University; Brown University

**Keywords:** Phosphatidylethanol, dried blood spots, timeline followback, AUDIT-C, men who have sex with men

## Abstract

**Background:**

Phosphatidylethanol (PEth), a phospholipid obtained from whole blood, offers superior sensitivity and detection windows over other biomarkers of alcohol use, and because it can be assayed from dried blood spots (DBS), it can be a suitable biomaker for virtual clinical trials. This study tested the feasibility of utilizing self-collected DBS to obtain PEth among men who have sex with men (MSM) in two virtual trials and examined associations between PEth and self-reported alcohol use.

**Methods:**

Participants with hazardous drinking were included from Ending the HIV Epidemic (EHE) jurisdictions in the US South and mid-Atlantic who enrolled in two virtual trials from 2021–2024. One trial focused on MSM who were taking HIV pre-exposure prophylaxis (PrEP), and the other was focused on MSM who were not. PEth was analyzed in DBS, and online surveys collected Timeline Follow-back (TLFB) and AUDIT-C data at baseline, three, and six months. Mixed-effects models evaluated predictors of DBS return rates. Spearman rank correlations assessed associations between PEth and self-reported alcohol use, and mixed-effects models examined moderators of this association.

**Results:**

A total of N=339 MSM were provided DBS collection kits. DBS return rates were 94% at baseline, declining to approximately 71–73% at follow-up. Fewer than 1% of samples were rejected for quality-related reasons. Agreement between PEth and self-report in classifying hazardous drinking across baseline and follow-ups was modest overall (62–66%). Spearman correlations were stronger for PEth and AUDIT-C (*r*s = 0.47) than PEth and TLFB (*r*s = 0.30).

PEth and self-report was consistently weaker in the trial where participants were on PrEP, with the pattern of results suggesting underreporting of alcohol use on self-report measures among those on PrEP.

**Conclusions:**

Self-collection of DBS for PEth analysis is feasible in virtual trials, although completion at follow-up remains challenging. Findings also suggested that brief self-report measures like the AUDIT-C may be the most practical complement to PEth in virtual alcohol research. Biomarkers should be incorporated into assessments of alcohol use in PrEP research, given that reliance on self-report measures may underestimate alcohol use.

## 1. Introduction

Alcohol use is the third leading preventable cause of death, accounting for 140,000 deaths in the United States (U.S.) (CDC, 2022; NIAAA, 2022b). Accurate assessment of alcohol use is essential for effective research and treatment but often relies on self-report measures that are susceptible to many limitations, including recall bias and social desirability bias (Latkin et al., 2017). The “gold standard” of self-report assessments are calendar-based methods such as the Timeline-Follow Back (TLFB) (Sobell and Sobell, 2000; Witkiewitz et al., 2015). However, these approaches are time-intensive and may introduce recall bias due to the difficulty in recalling past events (Carr et al., 2020; Merrill et al., 2020). A second widely used approach to assessing alcohol use, the Alcohol Use Disorders Identification Test – Consumption subscale (Bush et al., 1998), asks respondents to both recall and aggregate the frequency and typical quantity of drinking over a broad period of time, which could also introduce bias (Cherpitel et al., 2018; Gilligan et al., 2019).

Biomarkers can provide an objective assessment of alcohol use. Common biomarkers include breath analysis, Ethyl glucuronide (EtG; an alcohol metabolite detectable in urine), and liver enzymes (e.g., carbohydrate-deficient transferrin (CDT)). However, each of these approaches has limitations. Breath analysis can only detect recent drinking within hours before testing (Center for Substance Abuse Treatment, 2006); EtG also has a relatively small detection window (48–72 hours after consumption) and is prone to false positives (Helander et al., 2007), and liver enzymes are influenced by non-alcohol-related conditions (Harris et al., 2021; Neumann and Spies, 2003). Phosphatidylethanol (PEth), a direct blood biomarker, is a phospholipid that appears on the membranes of cells in proportion to blood alcohol concentrations. With a half-life of 4–10 days, PEth offers a longer detection window compared to urine metabolites like EtG and greater specificity than liver enzymes (Hahn et al., 2016a). Its diagnostic sensitivity for alcohol use is high. One study comparing different biomarkers found that PEth had 99% diagnostic sensitivity for detecting ethanol intake, surpassing the 40–77% sensitivity of common liver enzymes (Aradottir et al., 2006). Other studies have also consistently highlighted PEth's superiority as an alcohol biomarker (Neumann et al., 2020; Viel et al., 2012; Wurst et al., 2015). PEth can detect alcohol use up to 21 days post-consumption (Eyawo et al., 2020; Wurst et al., 2015), and differentiate between abstainers, moderate drinkers, and heavy drinkers (Helander et al., 2019; Piano et al., 2015; Ulwelling and Smith, 2018). However, some studies have indicated that PEth cannot distinguish between different types of alcohol consumption patterns, such as binge drinking versus daily consumption (Eyawo et al., 2020). Individual differences in alcohol metabolism may also affect PEth levels (Helander et al., 2019; Javors et al., 2016), but these variations are not correlated with demographic factors, which enhances PEth's validity across diverse populations (Viel et al., 2012).

Studies assessing the agreement between PEth and self-reported alcohol use have shown mixed results, with some studies finding relatively strong correlations (r = 0.50–0.80) (Cherrier et al., 2020; Hahn et al., 2012; Röhricht et al., 2020; Schröck et al., 2017; Skråstad et al., 2023) and others reporting more moderate-sized correlations (r = 0.20–0.40) (Richards et al., 2021; Wang et al., 2018). These discrepancies could be explained by a number of factors, including differences in study populations, methods for assessing self-reported alcohol use, individual’s personal characteristics, such as AUD history (Richards et al., 2021), and methods and procedures used to collect samples for PEth testing. A majority of studies comparing PEth values to self-reported alcohol use have used blood samples collected via venipuncture by trained professionals in face-to-face visits, which are then used to prepare dried blood spot (DBS) cards (Bakhireva et al., 2014; Faller et al., 2013). Others used slightly different approaches like finger-prick sampling, but still collected samples using trained professionals in face-to-face visits (Nwankwo et al., 2021).

More recently, the shift to remote research procedures has led to growing interest in whether patients can validly self-collect samples needed for biomarker analysis at home. Previous studies have established the feasibility and acceptability of DBS collected by patients at home and supported the validity of home-collected DBS for infectious disease testing (e.g., HIV, syphilis, HBV, HCV, SARS-CoV-2) (Karp et al., 2020; Loo et al., 2017; Miesse et al., 2022; Spielberg et al., 2000) and drug adherence monitoring (Linder et al., 2017). However, we are aware of only one small pilot study that explored the feasibility of self-collected DBS for PEth testing and it found that return rates were high at baseline (81%), but declined to 67–71% at follow-up (Firkey et al., 2023a).

Growing evidence suggests that accurately characterizing alcohol use in research requires combining self-report measures with biomarkers like PEth, rather than relying on either approach alone (Hahn et al., 2025). In traditional in-person research, self-report and biomarker collection can be closely synchronized, minimizing the gap in time between what a participant reports and what the biomarker captures. In virtual trials, this is more challenging; shipping delays, variable participant adherence, and the logistical complexity of coordinating remote procedures can cause surveys and biological samples to be collected days or weeks apart, during which time there could be a change in drinking behavior. Whether this variability meaningfully attenuates the correspondence between PEth and self-report, and whether it does so differently depending on which self-report measure is used, are important questions with direct implications for research design and assessment strategies.

In this study, we report findings on the feasibility of DBS self-collection for PEth assays in two virtual trials of men who have sex with men (MSM) who drank hazardously. Specifically, we explored rates of DBS sample return and whether demographic or behavioral characteristics were associated with the likelihood of returning samples. We also examined the concordance between PEth analyzed in self-collected DBS and self-reported data on alcohol use in the past 30 days by TLFB and AUDIT-C, and whether demographic or behavioral characteristics predicted concordance between PEth and self-report:.

## 2. Methods

### 2.1 Participants

MSM (*N* = 339) who reported hazardous drinking were recruited into two virtual clinical trials testing digital behavior change interventions using advertisements and organic posts on a variety of websites, social media, and gay-oriented online dating platforms. One study (NCT04973267) was focused on MSM who were on HIV pre-exposure prophylaxis (PrEP) medication (*N* = 93), and the other was focused on MSM who were not on PrEP (NCT04552171, *N* = 246). Full eligibility criteria for both studies are listed in Supplementary Table 1. All criteria were consistent across studies, with the exception of PrEP-related criteria. Both studies required that participants have home addresses in Ending the HIV Epidemic jurisdictions in the US South and mid-Atlantic. Analyses include participants who were enrolled in these trials between July 2021 and July 2024.

### 2.2 Measures

#### 2.2.1 Phosphatidylethanol (PEth) analysis.

US Drug Testing Laboratories (Des Plaines, IL) performed all PEth assays from DBS. Levels of the 16:0/18:1 species were quantified using standard liquid chromatography–tandem mass spectrometry (LC-MS/MS) protocols. The lowest quantity of the analyte that could be reliably quantified was 8.0 ng/mL. As HIV and STI testing were prioritized in the sample processing workflow, not all returned DBS samples had sufficient spots available for PEth analysis. In the study of MSM not on PrEP, DBS cards were returned to the HIV/STI testing laboratory, which was responsible for forwarding two spots to USDTL for PEth analysis. HIV and syphilis testing were intentionally prioritized, with available blood spots allocated to those assays first and remaining spots forwarded to USDTL when possible. This resulted in substantially fewer available PEth values for this study, particularly at the 6-month follow-up.

#### 2.2.2 Self-reported alcohol use.

Online Timeline Followback (TLFB): We used an online version of the TLFB procedure that assessed self-reported alcohol use in the 30 days preceding each timepoint (Sobell et al., 1996; Wray et al., 2019a). This tool first presented users with a calendar of the past 30 days and asked them to identify days on which they drank alcohol, which were marked with a “sticker.” Follow-up questions asked participants about their drinking behavior on that particular day. Participants reported the number of standard drinks they consumed each day (12 oz. beer, 5 oz. wine, 1.5 oz. liquor; a visual cue was included), as well as the total number of hours over which they drank. Past studies have suggested that computer-facilitated TLFBs for assessing alcohol use are reliable and valid (Hjorthøj et al., 2012; Pedersen et al., 2012; Simons et al., 2015; Sobell et al., 1996; Wray et al., 2019a).

Alcohol Use Disorders Identification Test (AUDIT): We used the AUDIT (Saunders et al., 1993) to characterize participants’ alcohol consumption in the past month, as well as participants’ risk for alcohol use disorder (AUD). The AUDIT is a 10-item survey assessing various symptoms and problems that are common in AUD, and is one of the most well-established measures of this construct globally (Dawson et al., 2005). We used the AUDIT – consumption subscale (AUDIT-C), which consists of the first three items of the AUDIT that assess alcohol use, to explore correlations between PEth values and aggregate self-report measures of alcohol use.

### 2.3 Procedures

In each study, interested participants who clicked on an ad or post were directed to a website that provided more information about the study. Participants then completed a brief screening that assessed basic eligibility criteria. If eligible, they were provided with details about each study and asked to provide informed consent and contact information. Staff then reached out to each registrant to verify information via phone or text message. Once confirmed, participants were considered enrolled, and research staff sent their first online survey via email and a DBS kit to participants’ confirmed home addresses. DBS kits contained: (1) a Whatman 903 Proteinsaver Card, (2) two alcohol pads, (3) two 2.0mm × 1.5mm high-flow, contact-activated lancets (BD; Franklin Lakes, NJ), (4) a sterile gauze pad, (5) two adhesive bandages, (6) a specimen bag pre-filled with (7) a silica gel-based desiccant pack, and (8) a postage-paid return envelope. Each kit came with written instructions that used simple infographics (Appendix A). Instructions also contained a QR code and URL to video instructions demonstrating how to collect DBS samples. Participants were encouraged to return their samples within two weeks of our original ship date. However, they were also encouraged to submit their samples at any time without penalty. Automated emails were sent to those who had not yet returned their samples a week after shipping, and every five days for a total of three reminders. Staff then called and texted those who still had not returned their samples after these reminders to encourage them to do so. Returned DBS samples were stored in a −30° C freezer and shipped in batches. We used a similar sequence of reminders to encourage completion of online surveys, which participants were prompted to complete quarterly via email. Participants in the PrEP study provided DBS samples and completed online surveys at baseline, 3 months, and 6 months, while participants in the non-PrEP study did so at baseline and 6 months. DBS kits were shipped to participants on or near the same day their follow-up surveys were sent. However, delays in shipping times and variability in participant adherence often led to these two procedures being completed at very different times, resulting in a “lag” between survey completion and DBS sample collection. Participants were paid $50–75 per kit and returned surveys, with incentives increasing at later follow-ups and bonuses provided for completing all required procedures.

### 2.4 Data analysis plan

We included all timepoints in which participants were sent sampling kits to maximize available data, regardless of whether participants were retained in their respective trials throughout follow-up. We first calculated overall rates of DBS sample return by assessment period. We then fit a Poisson mixed model of whether participants successfully returned a DBS sample at each timepoint to explore potential demographic and study-related predictors of adherence to this procedure. We used Poisson models because, although this outcome was binary (1 = DBS kit returned, 0 = DBS kit not returned), “success” was common at each timepoint (i.e., > 10%), which has been shown to produce biased estimates of odds ratios (McNutt et al., 2003; Nemes et al., 2009). This model used a log link function with robust standard errors. Coefficients were exponentiated to create risk ratios (RR) to aid in interpretation.

Next, we matched DBS sample collection dates with TLFB and AUDIT-C data, calculated summary statistics for the “lag,” and calculated Spearman rank correlations between the average drinks per week on the TLFB, AUDIT-C, and PEth values across various lengths of this lag. We then coded whether each of the focal measures, TLFB, AUDIT-C, and PEth, would suggest that participants drank at hazardous levels at that follow-up. For both TLFB and AUDIT-C, we coded follow-ups in which participants reported drinking ≥ 15 drinks in an average week in the past month or ≥ 6 drinks in a single occasion at least once in the past month as hazardous levels of alcohol use (NIAAA, 2022a). We coded PEth values of ≥ 50 ng/mL as similarly reflecting hazardous alcohol use (Hahn et al., 2021, 2016c). We then estimated the degree of agreement across each method and across various lag lengths (e.g., ≤ 7 days, 8–14 days, 15–21 days, 22–28 days, > 28 days).

Finally, to explore whether demographic and study-related factors were associated with correspondence between PEth values and each self-report measure, we estimated mixed effects regression models with PEth values as the outcome and self-reported alcohol use as a predictor, with interactions specified between self-reported alcohol use and each demographic and study-related predictor (e.g., age, follow-up interval). These interactions allowed us to test whether the relationship between PEth values and self-reported alcohol use varied across levels of each predictor. Given evidence of significant overdispersion in PEth values, we log-transformed PEth values prior to model fitting. We estimated separate models to assess correspondence between PEth and each self-report method (TLFB, AUDIT-C) and scaled and centered these self-reported drinking predictors to aid in interpretation of interaction effects. Both studies were approved by the local institution review board.

## 3. Results

Ninety-four percent (314/333) of sent kits were returned at baseline, versus 73.1% at Month 3 (57/78) and 71.3% at Month 6 (228/320). The final dataset included 463 total DBS samples that were matched to either TLFB or AUDIT-C (Table 1). Of the DBS samples received for PEth analysis, three were rejected, all due to having insufficient blood quantities for analysis.

Table 2 shows the results of a Poisson mixed model of returning a DBS sample at a given timepoint. Results showed that kit return rates were significantly lower at follow-up relative to baseline (*RR* = 0.77, *SE* = 0.06, 95% *CI* [0.66–0.91]), with about 23% fewer kits returned across both the 3- and 6-month intervals after accounting for covariates. We did not find that the rate of returning a DBS sample differed significantly across the two studies, or by participants’ age, employment status, or the severity of their alcohol use/problems.

Table 3 shows the number of successfully matched PEth samples with corresponding TLFB or AUDIT-C data, as well as agreement between PEth and each of the two self-report methods in classifying each follow-up as a hazardous drinking period. Agreement is also reported by the amount of “lag” between collecting each report or sample. Nearly half of DBS samples lagged online survey completion by 8–21 days, although 15–20% had lags of 28 days or more. Overall agreement was similar across the two methods: 66.3% of follow-ups characterized as involving hazardous drinking or not by PEth were also characterized similarly by AUDIT-C, compared to 61.8% for TLFB. Agreement did not appear to vary systematically with lag across either method, with no clear pattern of increasing or decreasing concordance as the interval between self-report and DBS collection increased.

Table 4 shows overall Spearman rank correlations between each method of assessing alcohol use, as well as by the length of lag between when each was collected. All methods were generally modestly correlated with each other overall, but with stronger associations between PEth-AUDIT-C (*rs* = 0.47) and AUDIT-C-TLFB (*rs* = 0.45) than for PEth-TLFB (*rs* = 0.30). Neither self-report method showed a clear trend toward weaker associations with greater lag lengths. PEth-AUDIT-C associations appeared remarkably stable over even longer lags.

**Figure 1:**
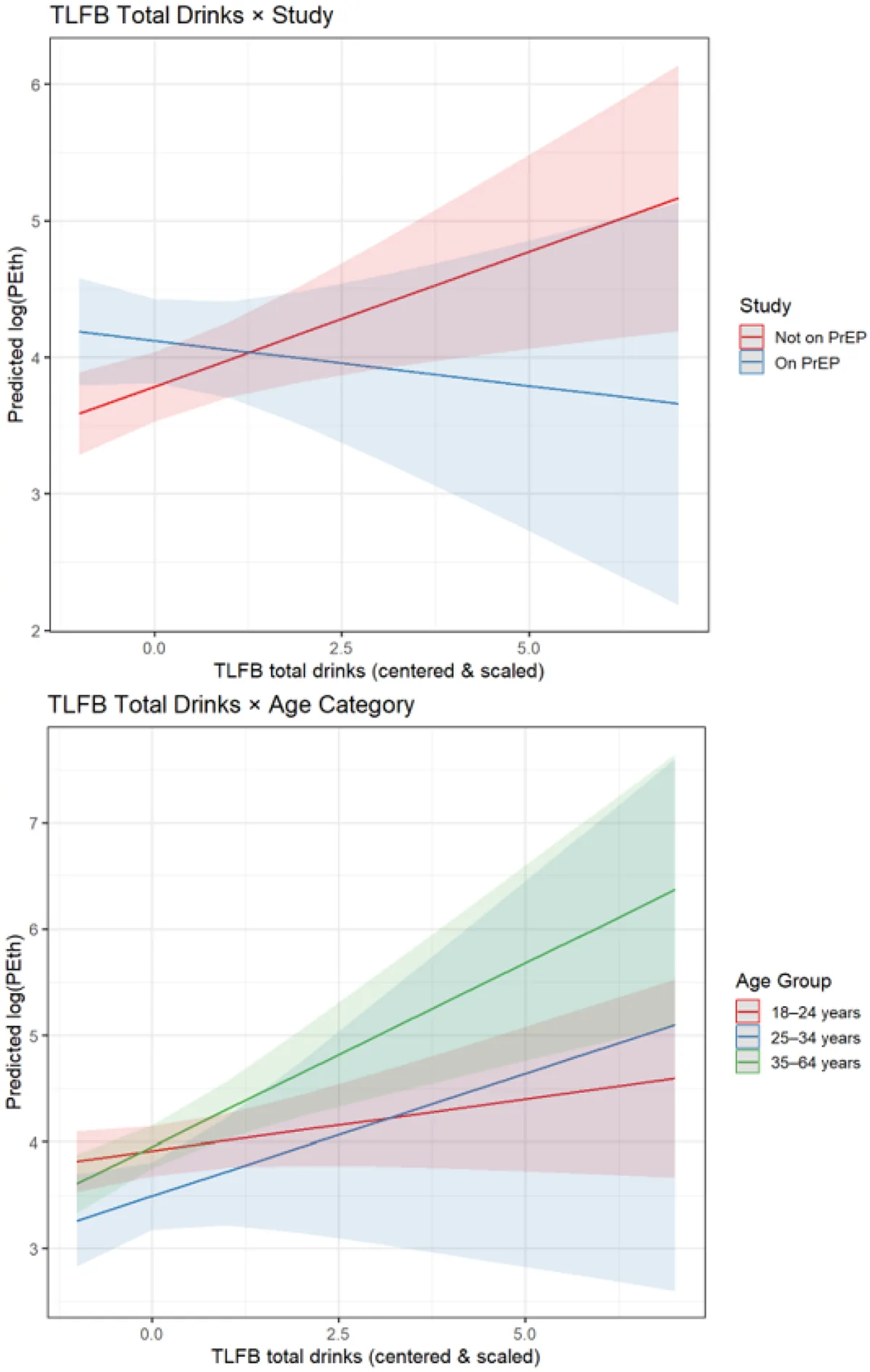
Model-predicted values of log PEth by total drinks reported on the Timeline Followback, parent study (upper) and age category (lower)

**Figure 2:**
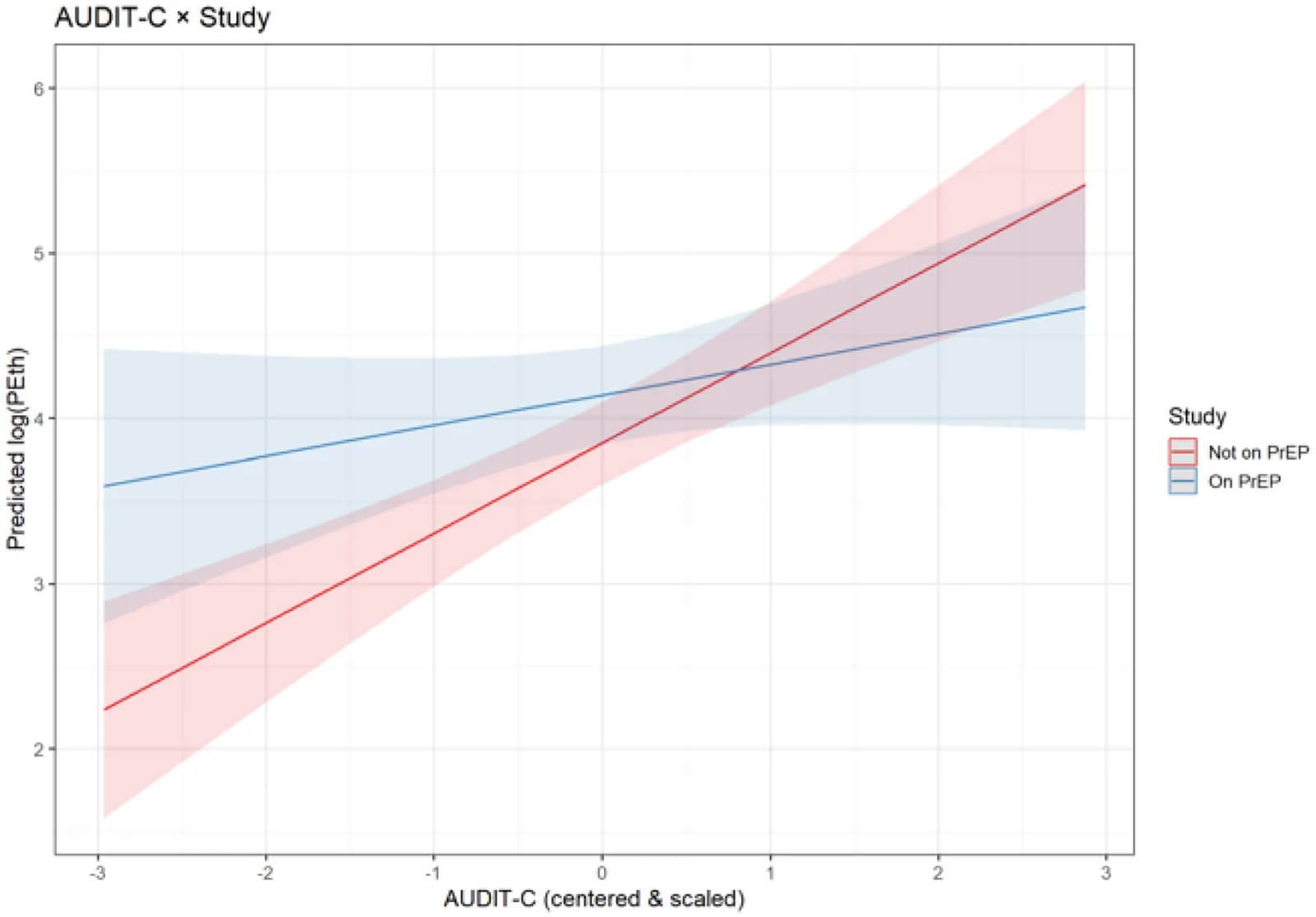
Model-predicted values of log PEth by AUDIT-C total score by parent study

**Figure 3:**
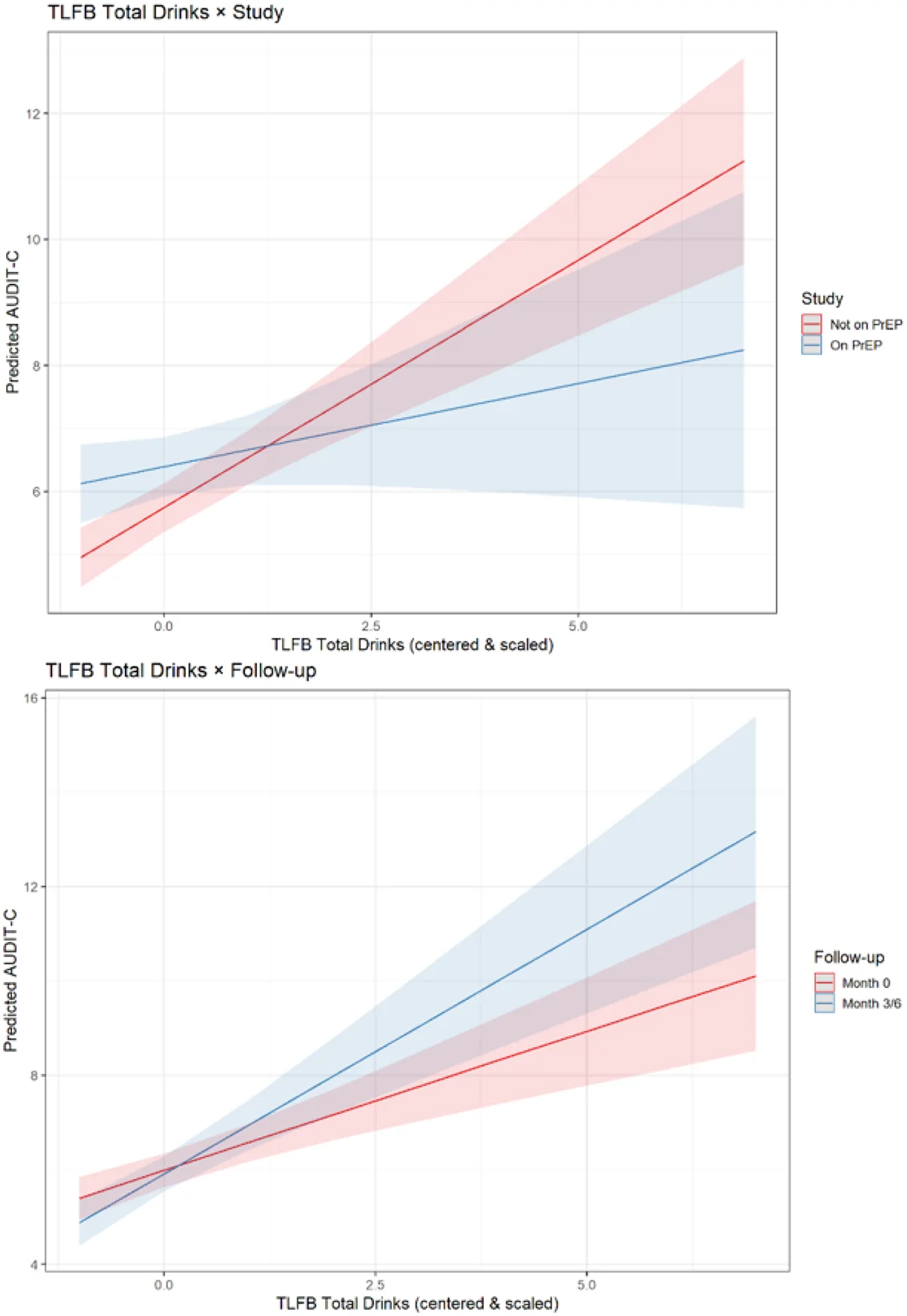
Model-predicted values of total alcohol drinks on the Timeline Followback by AUDIT-C total score, parent study (upper) and baseline vs. follow-up (lower)

Table 5 shows the results of linear mixed models of the correspondence between each method of assessing alcohol use. In the PEth x TLFB model (Model 1), the percentage of assigned surveys that participants successfully completed, a proxy for adherence to study procedures, was negatively associated with PEth values, suggesting that more engaged participants had meaningfully lower biomarker-detected alcohol exposure (β = −0.99, *SE* = 0.38 *p* = .009). A longer lag between self-report and DBS collection was also associated with modestly higher PEth values (β = 0.15, *SE* = 0.05, *p* = .002). A significant two-way interaction between TLFB and study suggested that the association between PEth and TLFB-reported drinks was significantly weaker in the study of MSM on PrEP than the study of MSM who were not on PrEP (β = −0.27, *SE* = 0.11, *p* = .017). The correspondence between PEth and TLFB was also stronger among 25–34 year olds, relative to the 35–64 reference group (β = 0.25, *SE* = 0.11, *p* = .023). See Fig. 1 for plots of interactions. The PEth x AUDIT-C model (Model 2) showed very similar results. A two-way interaction between AUDIT-C and study was significant (β = −0.36, *SE* = 0.12, *p* = .002) and suggested that the correspondence between PEth and AUDIT-C was also weaker in the PrEP study than the non-PrEP study (see Fig. 2). To test whether these effects were specific to PEth, we also fit a final model testing predictors of the correspondence between the two self-report methods (AUDIT-C x TLFB). A two-way interaction between TLFB and study predicting AUDIT-C scores was significant (β = −0.52, *SE* = 0.18, *p* = .005) and suggested that the correspondence between the two self-report methods was weaker in the PrEP study compared to the non-PrEP study. A significant two-way interaction between TLFB and follow-up (β = 0.45, *SE* = 0.17, *p* = .007) also suggested that the correspondence between TLFB and AUDIT-C was weaker at baseline than at follow-up (see Fig. 3).

## 4. Discussion

This study examined the feasibility of self-collected DBS for assessing PEth in two virtual trials with MSM who drank hazardously. We also explored the degree of correspondence between PEth and two self-report measures of alcohol use, the TLFB and AUDIT-C, and whether that agreement varied across demographic and study-related characteristics. We found that it is generally feasible for patients to self-collect DBS samples for PEth analyses, given that very few samples (< 1%) were rejected, all due to insufficient volume rather than improper preparation. These findings suggest that participants in virtual trials can adequately collect and return DBS samples when provided with clear written and video instructions, consistent with prior studies documenting high sample quality in remotely self-collected DBS (Firkey et al., 2023b; Hirshfield et al., 2018; Teran et al., 2021), although some exceptions have been noted (Nieuwenburg et al., 2024). Our findings extend this work by demonstrating feasibility in a larger virtual trial context, suggesting that PEth can be incorporated into remote alcohol research without resource-intensive collection infrastructure, potentially lowering barriers to its broader use in alcohol research.

There was moderate overall agreement between PEth and self-report measures, with PEth and AUDIT-C agreeing on 66% of follow-ups and PEth and TLFB agreeing on 62%. These levels of agreement are consistent with prior work documenting moderate correspondence between biomarker and self-report methods of assessing alcohol use (Hahn et al., 2012; Ferguson et al., 2020) and suggest that PEth and self-report may capture overlapping but distinct aspects of alcohol exposure. The fact that agreement between the two self-report methods themselves (64.8%) was similarly modest suggests that all three measures (TLFB, AUDIT-C, and PEth) capture alcohol use with some degree of error, and that discordance among them likely reflects measurement imprecision across all three approaches. This pattern supports the use of PEth alongside self-report rather than as a replacement, consistent with recommendations for combining biomarker and self-report assessment (Hahn et al., 2016b).

Spearman rank correlations provide further support for this general pattern. PEth and AUDIT-C correlations (*r*_s_ = 0.47) were somewhat stronger than PEth and TLFB correlations (*r*_s_ = 0.30), while AUDIT-C and TLFB were moderately correlated with each other (*r*_s_ = 0.45). Correlations between PEth and TLFB were weaker than those reported in several prior studies of men or mixed-gender samples, including a predominantly male outpatient sample (*r* > 0.60; (Helander et al., 2019)), people living with HIV (*r* = 0.67; (Ferguson et al., 2020)), and adolescents and young adults (*r*s = 0.70–0.75; (Röhricht et al., 2020)). Past research has shown that a variety of individual factors could influence PEth values, including timing and patterns of alcohol use (Hahn et al., 2012; Helander et al., 2019), differences in alcohol metabolism (Hahn et al., 2016a; Javors et al., 2016), and demographics like age, race, body mass index, or presence of AUD (Hahn et al., 2016a; Javors et al., 2016; Jorgenson et al., 2017). However, the virtual nature of the trials in the present study is likely a simpler explanation for the weaker correlations observed here than individual biological or demographic factors. Unsupervised, remote self-report could have yielded less reliable responses than interviewer-administered versions. The online version of the TLFB used here differs from the traditional interviewer-administered format in ways that may have made it easier for participants to underreport or speed through, attenuating its association with PEth relative to the AUDIT-C and potentially relative to what would be observed with a traditional TLFB. The stronger PEth-AUDIT-C association relative to PEth-TLFB is consistent with this explanation. The AUDIT-C, which captures more aggregated estimates of drinking patterns over time, may be more conceptually aligned with PEth as an integrative biomarker of cumulative alcohol exposure, whereas the TLFB's fixed 30-day recall window may not map as cleanly onto PEth's detection window.

Neither PEth-TLFB nor PEth-AUDIT-C correlations showed a clear pattern of attenuation with increasing lag between self-report and DBS collection, and PEth-AUDIT-C associations were notably stable even at lags exceeding 28 days. This finding is encouraging for researchers conducting virtual trials, where variability in the timing of sample and survey collection is difficult to avoid. It suggests that the correspondence between PEth and self-report is relatively robust to modest delays between the two, although replication in larger samples is needed.

We also tested whether the concordance between each assessment method varied across moderators such as demographic and study-related factors. Significant interactions between self-reported alcohol use and study emerged in all three models, suggesting that the correspondence between TLFB, AUDIT-C, and PEth was consistently weaker among participants in the PrEP study relative to the non-PrEP study. Importantly, this pattern held even in the model predicting AUDIT-C from TLFB, indicating that the weaker correspondence was not specific to PEth but reflected broader differences in self-report reliability between the two samples. One potential explanation is that, consistent with past studies of participants on PrEP and HIV treatment medications (Bajunirwe et al., 2014; Herrera et al., 2017; Jain et al., 2024), participants in the PrEP study may have underreported their drinking due concerns about how alcohol use might be perceived by researchers or fears that honest reporting could limit their access to prevention medications. Results also showed lower correspondence between the AUDIT-C and TLFB at baseline timepoints compared to follow-up. Although this could seem counterintuitive, attrition could be one potential explanation, given that those retained may be generally more conscientious and more likely to respond to surveys carefully, leading to higher correspondence between two self-report methods among those retained at follow-up.

In models predicting PEth values, survey completion rate—a proxy for adherence to study procedures—was negatively associated with PEth, which suggests that participants who were more consistently engaged with the study tended to have lower levels of biomarker-detected alcohol exposure generally. Researchers leading virtual trials should therefore be aware that retained, highly-engaged participants in their samples may not be representative of those with more severe alcohol use. A longer lag between collecting self-report measures of alcohol use and DBS collection was also associated with modestly higher PEth values in both models. Although it is surprising that lag was associated with PEth values directly rather than moderating its correspondence with self-report, one potential explanation for this finding is that it could simply reflect the fact that heavier drinkers took longer to return their kits, rather than any true effect of collection timing on the biomarker itself.

### Limitations

This study had important limitations. First, this study was focused exclusively on MSM, and enrolled largely White, non-Hispanic participants. Findings could differ substantially in other, more diverse populations with higher percentages of women or racial/ethnic minorities. Second, although DBS samples that were clearly inadequate were rejected prior to analysis, we could not independently verify that all returned samples were collected correctly at home. Samples still may have been improperly collected by, for instance, preparing blood spots that were incomplete or unevenly saturated. It is possible that samples like these may have been deemed usable but yielded artificially low PEth values, also weakening its correspondence with self-report. Third, our analyzed sample was limited to participants who both returned a kit and completed surveys at a given timepoint. Those who did not return kits or complete surveys may differ systematically from those who did in ways that could affect the generalizability of our findings.

#### Conclusions

In summary, we demonstrated that self-collection of DBS samples for PEth analysis is feasible in virtual trials, although sustaining kit return rates at follow-up dropped under 75%. Correspondence between PEth and self-report was modest and consistently weaker among participants in the PrEP study across all three models, suggesting that social desirability, potentially driven by concerns about how alcohol use might affect access to PrEP, could be an important source of error that researchers leading PrEP studies should be aware of. Correspondence between PEth and AUDIT-C was stronger than between PEth and TLFB, and was remarkably stable across varying lag lengths, suggesting that the AUDIT-C may be a better choice to complement PEth in virtual trials than TLFB.

## Supplementary Material

Supplementary Files

This is a list of supplementary files associated with this preprint. Click to download.

- SupplementaryInformation.docx

## Figures and Tables

**Table 1.** Demographics of participants who submitted at least one DBS sample by study

Characteristics	Not on PrEP (*N* = 246)	On PrEP (*N* = 93)
	Mean (*SD*) or *N* (%)	Mean (*SD*) or *N* (%)
Age (Range: 18 – 72)	32.8 (10.2)	34.7 (9.3)
Ethnicity (Hispanic or Latino)	49 (19.9%)	23 (24.7%)
Race		
White	178 (72.4%)	73 (78.5%)
Black or African American	30 (12.2%)	10 (10.8%)
Asian	13 (5.3%)	3 (3.2%)
American Indian/Alaska Native	3 (1.2%)	0 (0.0%)
Multiracial	16 (6.5%)	5 (5.4%)
Chose not to respond	6 (2.4%)	2 (2.2%)
College degree	163 (66.3%)	76 (81.7%)
Low income^1^	57 (23.2%)	12 (12.9%)
Unemployed	25 (10.2%)	3 (3.2%)
Sexual identity		
Gay	207 (84.1%)	85 (91.4%)
Bisexual	32 (13.0%)	7 (7.5%)
Straight	1 (0.4%)	0 (0.0%)
Other	4 (1.6%)	0 (0.0%)
Not sure	1 (0.4%)	0 (0.0%)
Chose not to respond	1 (0.4%)	1 (1.1%)
Baseline AUDIT total score		
Low Risk (1–7)	88 (35.8%)	21 (22.6%)
Harmful drinking (8–14)	90 (36.6%)	48 (51.6%)
High-risk (≥15)	68 (27.6%)	24 (25.8%)
Years on PrEP		2.5 (2.2)
Avg. days PrEP adherent/past 30		28.2 (3.6)

**Table 2.** Poisson mixed model of the rate of returning dried blood spot sample at baseline, follow-ups

Variable	*RR*	*SE*	95% *CI*	*p*
Intercept	1.04	0.17	0.75–1.42	0.826
*Time*				
**Follow-up**^1^	**0.77**	**0.06**	**0.66–0.91**	**0.002**
PrEP study^2^	0.87	0.08	0.72–1.05	0.143
Age (vs. 35–65)				
18–24	1.07	0.13	0.85–1.36	0.564
25–34	0.98	0.09	0.81–1.17	0.798
College education	1.11	0.11	0.91–1.36	0.305
Low income	0.98	0.11	0.79–1.23	0.876
Unemployed	1.06	0.17	0.78–1.44	0.708
AUDIT total score				
Moderate risk (8–14)	1.03	0.1	0.85–1.25	0.790
High risk (≥15)	0.96	0.11	0.77–1.20	0.732

Note.

1 Represents whether a given kit was collected at a follow-up timepoint (either 3 or 6 months) versus baseline.

2 Represents kits collected among participants in the study of MSM on PrEP, versus MSM not on PrEP.

**Table 3.** Timepoints meeting criteria for hazardous alcohol use by method and the number of days between when each method was collected

	TLFB^1^					AUDIT-C^2^		
Lag days^3^		PEth^4^ = 0	PEth = 1	Agreement		PEth = 0	PEth = 1	Agreement
Overall	TLFB=0	129 (27.9%)	88 (19.0%)	61.77% (n=286/463)	AUDIT=0	176 (38.0%)	41 (8.9%)	66.31% (n=307/463)
	TLFB=1	89 (19.2%)	157 (33.9%)		AUDIT=1	115 (24.8%)	131 (28.3%)	
≤7	TLFB=0	24 (31.2%)	12 (15.6%)	63.64% (n=49/77)	AUDIT=0	45 (39.5%)	9 (7.9%)	65.79% (n=75/114)
	TLFB=1	16 (20.8%)	25 (32.5%)		AUDIT=1	30 (26.3%)	30 (26.3%)	
8–14	TLFB=0	34 (26.6%)	28 (21.9%)	60.16% (n=77/128)	AUDIT=0	44 (41.1%)	13 (12.1%)	63.55% (n=68/107)
	TLFB=1	23 (18.0%)	43 (33.6%)		AUDIT=1	26 (24.3%)	24 (22.4%)	
15–21	TLFB=0	31 (31.3%)	20 (20.2%)	59.6% (n=59/99)	AUDIT=0	42 (43.3%)	8 (8.2%)	69.07% (n=67/97)
	TLFB=1	20 (20.2%)	28 (28.3%)		AUDIT=1	22 (22.7%)	25 (25.8%)	
22–28	TLFB=0	18 (28.6%)	13 (20.6%)	63.49% (n=40/63)	AUDIT=0	15 (34.1%)	3 (6.8%)	81.82% (n=36/44)
	TLFB=1	10 (15.9%)	22 (34.9%)		AUDIT=1	5 (11.4%)	21 (47.7%)	
>28	TLFB=0	21 (23.9%)	14 (15.9%)	64.77% (n=57/88)	AUDIT=0	20 (30.3%)	5 (7.6%)	60.61% (n=40/66)
	TLFB=1	17 (19.3%)	36 (40.9%)		AUDIT=1	21 (31.8%)	20 (30.3%)	

Note.

1 TLFB=Timeline Followback.

2 AUDIT-C=Alcohol Use Disorders Identification Test – Consumption scale.

3 Lag days= Represents the number of days between when participants provided self-report data about their alcohol use and when they prepared their dried blood spot cards. For example, if participants completed the TLFB on January 1 and collected their DBS PEth sample on January 9, “lag days” would be 9 and would fall in the 8–14 category.

4 PEth=phosphatidylethanol.

**Table 4.** Spearman rank correlations between phosphatidylethanol (PEth) in self-collected dried blood spots (DBS) and two survey methods of self-reported alcohol use

	Timeline Followback (TLFB)			AUDIT-C^1^		
Lag days^2^	*N*	Spearman ρ	*p*	*N*	Spearman ρ	*p*
≤7	77	0.216	.059	114	0.419	**<.001**
8–14	128	0.454	**<.001**	107	0.452	**<.001**
15–21	99	0.192	.057	97	0.516	**<.001**
22–28	63	0.347	**.005**	44	0.585	**<.001**
>28	88	0.233	**.029**	66	0.478	**<.001**

Note.

1 AUDIT-C=Alcohol Use Disorders Identification Test – Consumption scale.

2 Lag days= Represents the number of days between when participants provided self-report data about their alcohol use and when they prepared their dried blood spot cards. For example, if participants completed the TLFB on January 1 and collected their DBS PEth sample on January 9, “lag days” would be 9 and would fall in the 8–14 category.

**Table 5.** Mixed-effects models of correspondence between phosphatidylethanol (PEth) and self-reported alcohol use

	Model 1: PEth x TLFB				Model 2: PEth x AUDIT-C				Model 3: AUDIT-C x TLFB			
Predictor	*B*	*SE*	*p*	95% *CI*	*B*	*SE*	*p*	95% *CI*	*B*	*SE*	*p*	95% *CI*
TLFB/AUDIT-C	**0.89**	**0.33**	**.007**	**0.24–1.55**	**1.10**	**0.37**	**.003**	**0.38–1.82**	**1.88**	**0.54**	**<.001**	**0.83–2.94**
PrEP study^1^	**0.33**	**0.15**	**.028**	**0.04–0.63**	**0.29**	**0.15**	**.049**	**0.01–0.58**	**0.65**	**0.22**	**<.004**	**0.21–1.09**
Age 18–24 (vs 35–64)	**−0.42**	**0.20**	**.032**	−0.81–−0.04	**−0.52**	**0.19**	**.006**	**−0.89–−0.15**	0.47	0.29	.109	−0.11–1.05
Age 25–34 (vs 35–64)	0.04	0.15	.780	−0.26–0.34	−0.02	0.15	.899	−0.31–0.27	0.18	0.23	.438	−0.27–0.63
Follow-up (vs baseline)	0.10	0.07	.148	−0.04–0.25	0.11	0.07	.123	−0.03–0.26	−0.07	0.13	.569	−0.33–0.18
Survey completion (%)^2^	**−0.99**	**0.38**	**.009**	**−1.74–−0.25**	**−0.78**	**0.38**	**.041**	**−1.53–−0.03**	−0.39	0.58	.505	−1.52–0.75
Lag days^3^	**0.15**	**0.05**	**.002**	**0.06–0.24**	**0.11**	**0.05**	**.019**	**0.02–0.21**	**--**	**--**	--	**--**
Condition^4^	0.01	0.13	.936	−0.26–0.28	−0.07	0.13	.593	−0.33–0.19	0.03	0.20	.894	−0.37–0.43
TLFB/AUD × Study	**−0.27**	**0.11**	**.017**	**−0.48–−0.05**	**−0.36**	**0.12**	**.002**	**−0.59–−0.13**	**−0.52**	**0.18**	**.005**	**−0.89–−0.16**
TLFB/AUD × Age 18–24	0.13	0.19	.473	−0.23–0.50	0.01	0.15	.994	−0.30–0.30	0.41	0.31	.192	−0.21–1.02
TLFB/AUD × Age 25–34	**0.25**	**0.11**	**.023**	**0.03–0.47**	0.01	0.12	.924	−0.22–0.24	0.30	0.18	.105	−0.06–0.66
TLFB/AUD × Follow-up	0.10	0.10	.318	−0.09–0.29	−0.09	0.08	.255	−0.25–0.07	**0.45**	**0.17**	**.007**	**0.12–0.77**
TLFB/AUD × Survey %	−0.42	0.30	.159	−1.02–0.17	−0.20	0.35	.574	−0.88–0.49	−0.61	0.49	.214	−1.57–0.35
TLFB/AUD × Lag days	−0.04	0.05	.427	−0.16–0.06	0.04	0.05	.501	−0.07–0.14	--	--	--	--
TLFB/AUD × Condition	−0.07	0.10	.506	−0.26–0.13	−0.02	0.11	.851	−0.23–0.19	−0.02	0.17	.897	−0.35–0.31

Note.

1 Represents kits collected among participants in the study of MSM on PrEP, versus those collected in the study of MSM not on PrEP.

2 Represents the percentage of study surveys that participants completed, a marker of overall adherence to study procedures.

3 Represents the number of days between when participants provided self-report data about their alcohol use and when they prepared their dried blood spot cards. A higher “lag” means that more days elapsed.

4 Represents participants who were assigned to the intervention condition in either of the larger studies. The intervention in both studies involved different versions of the Game Plan app (Wray et al., 2024, 2019b).
